# Design of Soft Nanocarriers Combining Hyaluronic Acid with Another Functional Polymer for Cancer Therapy and Other Biomedical Applications

**DOI:** 10.3390/pharmaceutics11070338

**Published:** 2019-07-15

**Authors:** Marlène Rippe, Vanina Cosenza, Rachel Auzély-Velty

**Affiliations:** Grenoble Alpes University, Centre de Recherches sur les Macromolécules Végétales (CERMAV)-CNRS, 601, rue de la Chimie, BP 53, CEDEX 9, 38041 Grenoble, France

**Keywords:** hyaluronic acid, polymeric nanocarriers, drug delivery

## Abstract

The rapid advancement in medicine requires the search for new drugs, but also for new carrier systems for more efficient and targeted delivery of the bioactive molecules. Among the latter, polymeric nanocarriers have an increasingly growing potential for clinical applications due to their unique physical and chemical characteristics. In this regard, nanosystems based on hyaluronic acid (HA), a polysaccharide which is ubiquitous in the body, have attracted particular interest because of the biocompatibility, biodegradability and nonimmunogenic property provided by HA. Furthermore, the fact that hyaluronic acid can be recognized by cell surface receptors in tumor cells, makes it an ideal candidate for the targeted delivery of anticancer drugs. In this review, we compile a comprehensive overview of the different types of soft nanocarriers based on HA conjugated or complexed with another polymer: micelles, nanoparticles, nanogels and polymersomes. Emphasis is made on the properties of the polymers used as well as the synthetic approaches for obtaining the different HA-polymer systems. Fabrication, characterization and potential biomedical applications of the nanocarriers will also be described.

## 1. Introduction

In recent years, advances in nanotechnology have provided researchers with new diagnostic and therapeutic tools. Among the latter, nanocarriers, i.e., carriers having typically submicron sizes of about 20–250 nm and forming a separated phase in aqueous media [1], have been one of the most important tools, as they offer solutions to overcome many problems of the actual therapies. One of the main advantages of these nanosystems is their possibility to carry drugs (chemotherapeutic agents, biological agents, immunotherapeutic agents etc.) to the desired sites of therapeutic action while reducing adverse side effects. They also offer additional advantages, such as enhancing the aqueous solubility of poorly soluble drugs, protection of drugs from degradation or instability in biological medium and helping the biodistribution of the drug [2]. Furthermore, due to their size, it is possible to work in a scale comparable to proteins, DNA, organelles, etc., thus being capable of interacting with them on both the surface and inside cells [1,2]. Cell recognizing ability of nanocarriers is usually achieved by the addition of biomolecules like proteins, peptides, oligo- or polysaccharides to the nanosystem, either by decoration of its surface or as a component of the shell. Moreover, for anticancer therapy, nanocarriers are capable of passive accumulation in tumors because of the enhanced permeability and retention (EPR) effect due to the high leakiness of tumor blood vessels [3].

Nanocarriers can be composed of different inorganic and/or organic materials. Among the former, quantum dots, gold and iron oxide nanocarriers have been used for imaging, diagnostics and drug delivery [4]. However, these types of carriers suffer from limitations, notably low drug loading capability and potential toxicity [5,6]. With regard to nanocarriers based on organic compounds, one of the best-known examples is liposome: vesicle in which an aqueous volume is enclosed by a membranous lipid bilayer [2]. This type of nanocarrier is biocompatible, and, due to its amphiphilic character, is capable of encapsulating both hydrophobic and hydrophilic drugs. Another class of organic-based nanocarriers consists of polymeric nanosystems, that can be made of synthetic and/or natural polymers. The main advantage of these nanocarriers is that, depending on the choice of polymer, it is possible to tailor the physical, chemical and biological properties of the nanosystems, thus giving a large spectrum of possible nanovehicles for drug delivery applications [5]. At first polymeric nanocarriers were based on non-biodegradable polymers, however, systems should be designed so they do not accumulate in tissues at toxic levels [7]. For this reason, biodegradable polymer-based nanocarriers became of interest to avoid potential toxicity problems. Nevertheless, they are far from optimal with respect to drug release profile: a considerable amount of drug is usually released upon injection as a result of inadequate stability while the drug cannot readily be released from nanocarrier following their arrival at the pathological sites. To circumvent these limitations, stimuli-responsive polymeric nanoparticles became the focus of considerable interest as a result of their on-demand drug release ability as well as their tunable physicochemical properties. Besides, nowadays, many researches have been directed towards the use of natural polymers, like polysaccharides, for the design of nanovehicles for drug delivery, due to their unique physical and chemical properties which make them a special class among polymer materials. They are generally biocompatible and biodegradable and may also exhibit a biological activity which can be advantageously exploited for engineering drug delivery systems. This is highlighted in the case of hyaluronic acid (HA), which belongs to the family of glycosaminoglycans (GAGs). This is a linear polysaccharide composed of d-glucuronic acid and *N*-acetyl-d-glucosamine repeating units. HA is a major component of the extracellular matrix (ECM) of biological tissues where it plays major structural and biological roles [8]. In particular, this polysaccharide can be specifically recognized by membrane receptors (such as CD44) present on most mammalian cells and many cancer cells. For these reasons, HA-based polymeric nanocarriers have been largely investigated as therapeutic tools, mainly for anticancer therapy, but also for other biomedical applications.

This review describes a comprehensive outline of diversified nanocarriers based on HA conjugated or complexed with another polymer. For ease of reading, these systems will be referred to as HA-polymer conjugates or complexes, respectively, throughout the text. We have deliberately kept our focus on HA-polymer nanocarriers and self-assemblies such as solid nanoparticles, nanogels, polymersomes and micelles as well as the main approaches that have been undertaken to design these hybrid nanosystems. We also explore how the combination of HA and the polymer in the nanocarrier influences its use as a drug delivery system for therapeutic or imaging agents.

## 2. Types of Polymeric Nanocarrier Systems Based on HA

**Figure 1:**
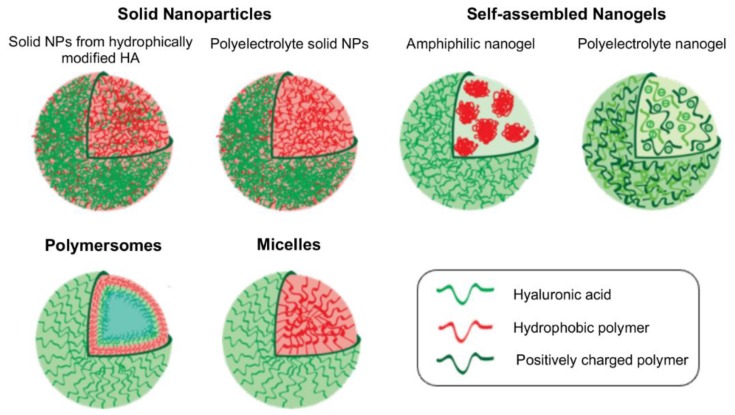
Schematic representation of different hyaluronic acid (HA)-based polymeric nanocarriers.

A wide variety of nanosized polymeric carriers based on HA, with distinct architectures, sizes and surface properties have been developed. These HA-based nanocarriers can be classified depending on the properties of the grafted synthetic polymer (hydrophobic, hydrophilic or amphiphilic character), the architecture of the HA-polymer hybrid (block copolymer, graft copolymer or polyelectrolyte complex) and whether it self-assembles or requires a fabrication process. In this way, it is possible to obtain solid nanoparticles, nanogels, polymersomes and micelles. Herein, HA-decorated composite nanoparticles are not included as polymeric nanocarriers since the polysaccharide is mainly located on the carrier surface and is not the major component. An illustration of the different types of HA-based polymeric nanocarriers is given in Figure 1.

### 2.1. Solid Nanoparticles

Solid polymeric nanoparticles are nanosized particles made of hydrophobic polymers, which can be neutral or charged [1]. These nanoparticles have high hydrophobic drug-loading capacity; however, they also suffer from rapid removal by macrophages as well as cytotoxicity issues. For this reason, it is important to add biocompatible peptides or polysaccharides, like HA.

HA-based polymeric nanoparticles are mainly obtained using neutral polymers. These nanoparticles are frequently fabricated via a physical method, i.e., polymers are first synthetized and then, nanoparticles are obtained by an external process (dialysis, solvent evaporation, nanoprecipitation, sonication, etc.) applied to the pre-formed HA-polymer conjugate [2,9]. The selection of the process defines the size and polydispersity of the nanoparticles. Examples of HA-based nanoparticles from charged polymers, i.e., “polyelectrolyte solid nanoparticles”, can be also found in literature [10,11]. In this case, nanoparticles are formed by complexation of HA with a positively charged hydrophobic polymer. For this type of nanoparticle, a chemical method is generally preferred for their fabrication. Indeed, the nanocarrier is obtained during the synthesis of the synthetic polymer, and HA is used as a stabilizer of the ionic monomers and finally coats the hydrophobic polymer by complexation (surfactant-free emulsion polymerization method) [9,10,11].

### 2.2. Nanogels

The ‘‘nanogel’’ term was first introduced to define crosslinked bifunctional networks of a polyion and a nonionic polymer for delivery of antisense oligonucleotides [12]. These nanocarriers are nanosized three-dimensional networks of chemically or physically crosslinked hydrophilic polymers [13,14,15,16,17,18,19]. It should be pointed out, that in some cases physically crosslinked nanogels can be chemically crosslinked, in a second step, to improve their long-term stability when circulating in the bloodstream [20].

Chemically crosslinked nanogels are obtained by formation of covalent bonds between hydrophilic polymers, either during polymerization or in a second step, from pre-formed polymers [16]. HA-based nanogels are prepared by the second method, based on the crosslinking of HA with small molecular crosslinkers and the use of water-in-oil emulsions as nanoreactors [13,21].

Physically crosslinked nanogels can be formed through various physical interactions, including hydrogen bonding, electrostatic interactions and hydrophobic interactions. These nanogels can therefore be divided into two main groups whether they are obtained by polyelectrolyte complexation of oppositely charged polymers or by self-assembly of amphiphilic polymers. Since HA is negatively charged, complexation strategy with oppositely charged polysaccharides or polymers, like chitosan (CS) [22], poly(l-lysine) [23], poly(β-amino ester) [24] has been used for the formation of “polyelectrolyte nanogels”. However, the hydrophilic nature of these nanosystems makes the encapsulation of neutral hydrophobic drugs impossible. On the other hand, nanogels made of amphiphilic polymers are capable of encapsulating small hydrophilic as well as hydrophobic molecular therapeutics, biomacromolecules and inorganic nanoparticles within their crosslinked network.

Compared to other nanoparticles, nanogels are able to take up large amounts of water or physiological fluid, while maintaining their internal network structure. Thus, they exhibit the greatest flexibility and deformability, high loading capacities (30 to 50% weight) [18] and the particular feature of efficiently loading drug at any time, i.e., when the nanogels are swollen and equilibrated in water or in biological fluid.

### 2.3. Polymersomes

Polymersomes, in analogy to the lipid-based vesicles, liposomes, [25,26] are hollow spheres consisting of a polymer bilayer in which both hydrophilic and hydrophobic therapeutic molecules can be encapsulated in the aqueous core and in the polymer shell, respectively. The bilayer is made from amphiphilic diblock (A-B) or triblock (A-B-A) copolymers [27]. The size and morphology can be controlled by varying the composition and size of the copolymer and fabrication methods (like solvent exchange, double-emulsion, etc.). Compared to liposomes, polymersomes have a higher membrane thickness, which provides to the bilayer higher stability and robustness against mechanical stresses [25]. Moreover, delivery of two different kinds of drugs can work synergistically to enhance the therapeutic effect. So far, however, very few studies have been reported on polymersome formation from HA-based diblock copolymers [28,29,30,31].

### 2.4. Micelles

Similar to polymersomes, micelles are composed of amphiphilic block copolymers. The difference between them lies in the architecture of the self-assembled particle. Micelles are characterized by amphiphilic block copolymers adopting, in aqueous solution, a core-shell structure that can directly entrap the cargo in the hydrophobic core [32]. Thus, only hydrophobic compounds can be encapsulated. The two major advantages of these structures are that (i) the synthesis of block copolymer enables the design of well-defined and controlled architectures, (ii) they allow to preserve fully or to a large extent the integrity and properties of the HA backbone.

The transition from polymersomes to micelles and vice-versa is driven by the hydrophilic/hydrophobic balance. Depending on the ratio of the hydrophilic part to the total mass of the copolymers, it is possible to obtain polymersomes (when the hydrophilic content is <40% weight of the polymer molecule) or spherical micelles (>50%) [25].

## 3. Synthetic Strategies for the Design of HA-Based Nanocarriers 

**Figure 2:**
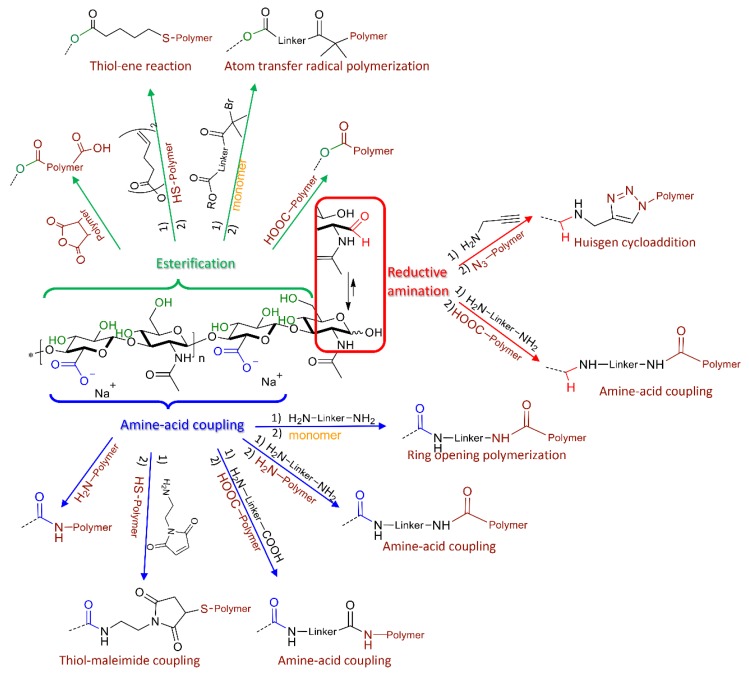
Different strategies for coupling synthetic polymers with HA via its carbonyl group (red), carboxylate groups (blue) and hydroxyl groups (green).

Different strategies for the generation of nanocarriers have been designed depending on whether a polyelectrolyte complex or a conjugate (either block or graft copolymer from HA) is desired. For the latter case, there are three types of functional groups in HA that can be used for the coupling: the anomeric carbonyl group, the hydroxyl groups or the carboxyl groups (Figure 2). Depending on the targeted group of HA and the functional groups in the synthetic polymer, the conjugates can be obtained by a direct reaction between both macromolecules, or, in most cases, a modification of HA and/or of the synthetic polymer is required as a preliminary step to incorporate new functional reactive groups. This modification is usually performed by adding a linker that may also provide other properties to the nanocarrier (like redox sensitivity if the linker contains a disulfide bond). The different strategies are described in this section.

### 3.1. Block Copolymers from HA

Amphiphilic block copolymers (HA-*b*-polymer, Table 1) are synthesized by end-to-end coupling strategies. This is achieved by modification of the terminal reducing end of HA, which is in equilibrium between the hemiketal and the aldehyde form (Figure 2). The aldehyde form can react with amine-functionalized molecules by a reductive amination reaction to add a new functional group. The most common strategy relies on the addition of an alkyne group to perform a Huisgen cycloaddition with an azide-containing polymer. An alternative synthetic approach is to modify the carbonyl group with a diamine and then graft the polymer via amide bond formation between the polymer and the free amine group [33].

### 3.2. Graft Copolymers via “Grafting onto” or “Grafting from” HA

Two different strategies can be used to obtain HA-*g*-polymers summarized in Table 2. The most frequently used approach is the “grafting onto” method that requires the preliminary synthesis of end-functionalized polymers that are subsequently covalently bonded to the polysaccharide. The advantage of this technique is that it allows synthesis of the polymer in optimal conditions before grafting. However, due to steric hindrance, low grafting degrees are frequently observed [38]. The other strategy is the “grafting from” method, in which the polymer chain “grows” from the HA backbone. This approach involves polymerization methods such as the ring-opening polymerization (ROP), controlled radical polymerizations (CRP), or oxidative polymerizations [39]. Functional groups in HA, or previously modified HA, act as initiating species for the polymerization [40]. However, the main limitation of this technique is that polymerization has to be carried out in water.

Regardless the grafting technique, HA-*g*-polymers are obtained via conjugation on the carboxylic acid or hydroxyl groups of HA (Figure 2). Thus, multiple attachments occur, and the synthetic polymers are randomly linked to the polysaccharide backbone. Nonetheless, a high degree of substitution (DS, average number of substituting groups per repeating disaccharide unit) can affect the biodistribution of HA as well as its interaction with protein receptors. For instance, when the carboxylate group is selected, it has been found that a DS above 0.25 decreases the ability of HA to target CD44 receptors [41].

Coupling via carboxylic groups is generally carried out via amine-acid coupling and esterification reactions. The main strategy is based on amide bond formation using carbodiimide-mediated coupling reactions (Figure 2). Various amine-functionalized polymers have been grafted onto HA through the carboxylic acid group. Moreover, it can also be used to add new functional groups like maleimide [20,42,43] or amines [39,44,45,46]. Functionalization with a maleimide is useful for grafting thiol-containing polymers. Addition of an amine group allows a second amine-acid coupling with carboxylate bearing polymers. Furthermore, amine-modified HA can be used as initiator of ROP polymerization [39].

The hydroxyl groups are commonly converted into ester or ether derivatives, the former being the most commonly used route (Figure 2) [47]. Direct esterification of hydroxyl groups of HA can be carried out by acid-bearing or anhydride-bearing polymers. Furthermore, modification of this group with linkers containing another functional group has also been done. The reaction of HA with pentenoic anhydride allows the introduction of alkene groups, which can subsequently react with a thiol-containing polymer via a radical thiol-ene reaction [48,49]. Another strategy consists in adding a linker containing 2-bromo-2-methyl propionic acid that acts as an initiator for atom transfer radical polymerization (ATRP), through the “grafting from” strategy [40].

### 3.3. HA-Polymer Complexes

Since HA is negatively charged at physiological pH, its complexation with positively charged polymers has been frequently used for the synthesis of HA-based nanocarriers. As mentioned in Section 2, this complexation can lead to nanogels or nanoparticles depending on the solubility of the polymer. These nanocarriers are listed in Table 3.

## 4. Nanocarriers Based on HA-Polymers Conjugates: Biomedical Applications

A large variety of HA-based polymeric nanocarriers can be found in the literature. As previously mentioned, the scientific interest in using HA as a building block for nanocarrier design is related to the biocompatibility, biodegradability of HA and its ability to target CD44-overexpressing cancer cells. Nonetheless, the choice of the synthetic polymer that is conjugated to HA is also an important issue. As described in Section 2, depending on the properties of the polymer (hydrophilic, hydrophobic or amphiphilic), different types of nanocarriers are obtained. Furthermore, the synthetic strategy for coupling the polymer to HA is usually selected depending on the functional groups displayed by the former. Moreover, the synthetic polymers can also provide new properties to the nanocarriers. For instance, biodegradable polymers are usually the preferred option as they confer to the nanocarriers complete degradability, allowing them to be excreted [61]. Charged polymers are selected when complexation with an oppositely charged molecule is desired, thus generating polyelectrolyte nanocarriers. This could be used for crosslinking with HA negative charges (as described in Section 2.1 and Section 2.2) or for encapsulating a charged molecule by complexation. One of the most common applications of the latter is the delivery of negatively charged siRNA or miRNA. Nanocarriers are designed by grafting or blending the polysaccharide with positively charged polymers. Another strategy reported in the literature for designing HA-based nanocarriers is based on the modification of HA with stimuli-responsive polymers (pH-responsive, thermo-responsive, redox-responsive or light-responsive). Delivery systems based on these polymers are capable of altering their chemical and/or physical properties upon exposure to external stimuli. The observed changes are mainly disassembly, decomposition, activation of supramolecular aggregation in response to a specific cellular/extracellular stimulus of chemical, biochemical, or physical origin, resulting in the release of the active species to specific biological environment [62,63]. In this regard, selective drug release by pH- and redox-responsive nanocarriers can be achieved due to the difference in pH and reducing environment between normal and cancer cells. Thermoresponsive nanocarriers allow self-assembly of polymeric systems at body temperature while being soluble at lower temperature. Light-responsive nanocarriers are dependent on external light source. Thus, the desired effect only happens when and where light is applied. In this section, a brief compilation of the different HA-based polymeric nanocarriers is presented with emphasis on the properties of the nanocarriers and their biomedical application (see Table 1, Table 2 and Table 3).

### 4.1. Biodegradable Nanocarriers

A biodegradable polymer may be described as a polymer which degrades through the cleavage of covalent bonds via a chemical process (hydrolysis, or oxidative and enzymatic mechanisms) [64]. This type of polymer has been largely used for biomedical applications as it does not require removal or further treatment after introduction into the body [61]. Biodegradable polymers can be classified according to their degradation process: hydrolytically degradable polymers (i.e., polyesters, polycarbonates, polyphosphazenes, polyphosphoesters, etc.) and enzymatically degradable polymers (mainly proteins, poly(amino acids) and polysaccharides).

Among the hydrolytically degradable polymers, the most frequently used for the synthesis of the HA-based nanocarriers are polyesters like polycaprolactone (PCL) [31,34,39], poly(lactide) (PLA) [50], and poly(lactide-*co*-glycolide) (PLGA) [33,44,45,46,51,52] (Table 1 and Table 2). Nonetheless, other biodegradable polymers like polycarbonates have also been used for the synthesis of HA-based nanocarriers [36].

Hass et al. [31] and Han et al. [34] synthesized HA-*b*-PCL via Huisgen cycloaddition to obtain polymersomes and micelles, respectively. The polymersomes were produced using the solvent-shift method with chloroform and water, which yielded particles in a size range of 50−400 nm. As expected, they were capable of encapsulating both hydrophilic (in the core) and hydrophobic (in the shell) dyes. The authors demonstrated that the size distributions and the hydrodynamic diameter varied considerably depending on the cargo molecule without following a clear trend. On the other hand, the micelles were obtained by self-assembly in water and exhibited spherical shape and unimodal size distribution. However, only hydrophobic drugs, like doxorubicin (DOX), could be encapsulated. 

Agrawal et al. designed nanoparticles by nanoprecipitation after grafting PLGA onto HA, using a PEG diamine as a linker [46,52]. These nanocarriers were capable of encapsulating different hydrophobic drugs like DOX and 5-fluorouracil (5-UF) (Table 2). The authors observed that the drug-loaded nanoparticles based on HA-PEG-PLGA were more concentrated in the tumor, thus causing a more significant reduction of the tumor, than the drug alone or the drug-loaded nanoparticles based on PEG-PLGA. Furthermore, biodistribution studies showed that these HA-based nanoparticles are efficient carriers of hydrophobic drugs with reduced accumulation in non-tumor tissue.

**Figure 3:**
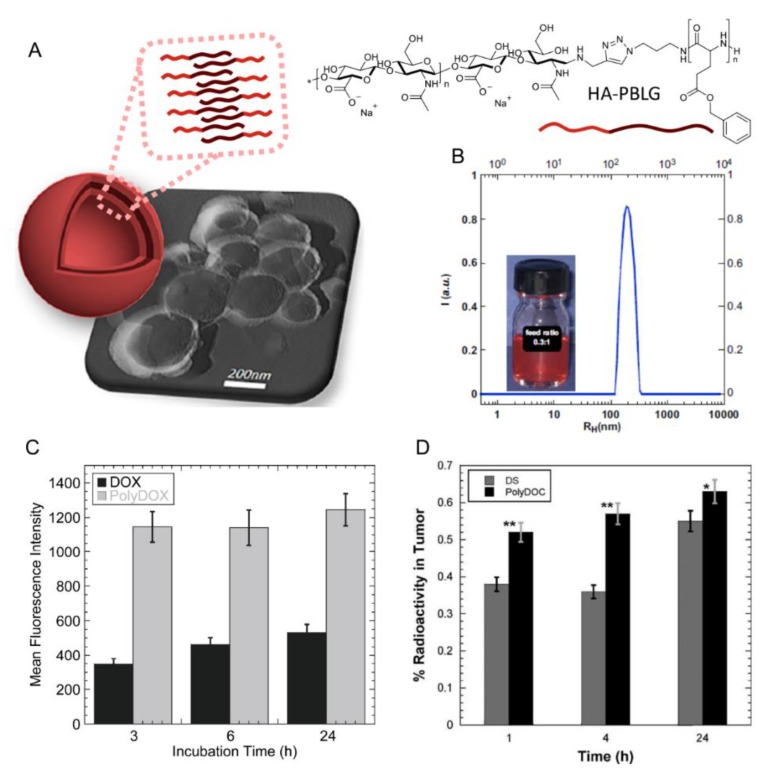
(**A**) Schematic representation and TEM imaging of the prepared polymersomes. (**B**) DLS size distribution of DOX loaded polymersomes (PolyDOX). Insert is a photo of a PolyDOX solution (**C**) represent DOX and PolyDOX uptake in MCF-7 cells at 10 mM DOX concentration (**D**) Tumor uptakes of ^99m^Tc labelled-DOC solution (DS) and ^99m^Tc labelled-PolyDOC (both at 2 mg/kg mice). Each point represents the mean of three mice ± S.D. (* *p* < 0.05 vs. DS, ** *p* < 0.001 vs. DS). The results are expressed as the mean ± SD (*n* = 3). Adapted with permission from [28,29]. Copyright 2019, John Wiley and Sons and Elsevier, respectively.

Poly(amino acids) are the most common enzymatically degradable polymers which are conjugated to HA for the fabrication of nanocarriers [28,29,30,35,57,58,59,60]. Lecommandoux et al. used poly(benzyl-l-glutamate) (PBLG) for the synthesis of HA-based polymersomes (Figure 3) with a size around 200 nm to convey different chemotherapeutic drugs such as DOX [29,30] and docetaxel (DOC) [28], with a high drug loading capacity (see Table 1). These polymersomes accumulated at the tumor site in addition to liver, lungs and spleen. DOC-and DOX-loaded HA-b-PBLG_60_ (PolyDOC and PolyDOX respectively) uptake in tumor was larger at each time (1, 4 and 24 h) compared to the free drugs. Lastly, by varying the hydrophilic/hydrophobic balance of the copolymer and the fabrication method, the authors were able to obtain nanoparticles of different sizes (300 or 30 nm) [35]. The authors observed that size plays a role in cellular uptake in vitro. Nanoparticles of 30 nm were bound and internalized by non-small cell lung cancers cells or NSCLC (cell lines H322, H358 and A549) more efficiently (2−3 times higher) than NPs of 300 nm. However, in vivo studies could not confirm these results. After intravenous administration, both systems have the same ability to reach the tumor sites for H358-bearing mice. For A549-bearing mice, a slight preferential uptake of 30 nm-size polymersomes was detected, but mostly were rapidly taken by the liver after administration.

### 4.2. Polyelectrolyte Nanocarriers

Polyelectrolytes nanogels have been obtained by blending HA with chitosan [22], poly(l-lysine) [23] and poly(β-Amino Ester) [24] (Table 3) or by conjugation of HA with poly(ethyleneimine) (PEI) [54,55,56] and with poly(*N*,*N*-diethylaminoethylmethacrylate) (pDEAEMA) [40] (Table 2). The use of these amphoteric positively charged polymers has a second advantage as the nanogels can encapsulate negatively charged molecules like miRNA or siRNA that can elicit RNA interference once in the target cell, thus silencing the desired gene. The difference between miRNA or siRNA is that the former is designed to specifically knock down a single gene, while the latter may target multiple different genes [22]. The interest in designing nanocarriers to deliver siNRA or miNRA lies in the fact that naked molecules are not suitable for systemic delivery due to their size, negative charge and degradability by serum endonucleases [54]. For this reason, Palumbo et al. [40] prepared HA-pDEAEMA nanogels for delivery of Luciferase GL3 duplex siRNA. The authors synthesized the nanogels by ATRP polymerization of diethylaminoethyl methacrylate (DEAEMA) from HA modified with 2-bromo-2-methylpropionic acid. The ratio of HA: DEAEMA defines whether the nanogels has a positive or negative ξ-potential. Nonetheless, in both cases, siNRA can be encapsulated. Gene-silencing efficiency of the encapsulated siNRA was confirmed by in vitro experiments. It can range from 40−90% depending on the dose and time. Moreover, it was observed that the cell uptake is due to CD44-mediated endocytosis. Deng et al. [22] designed HA-CS nanocarriers via ionotropic gelation for encapsulation of miR-34a (one of the most studied tumor suppressor miRNA) and DOX. Given the opposite charge of both polysaccharides, a primary ionic interaction induces the formation of polyelectrolyte nanogels. Furthermore, the negative charge of miRNA and positive charged of DOX favor the encapsulation of both drugs. Crosslinking of these nanogels is achieved by addition of tripolyphosphate during the ionotropic gelation process. The size of the nanogels (160−220 nm) varies depending of the ratio of HA:CS as well as if the drugs are encapsulated or not. In vitro and in vivo assays revealed a synergistic effect of both drugs where miR-34a was capable of enhancing DOX antitumor activity by silencing the expression of the anti-apoptosis proto-oncogene Blc-2. Furthermore, this miRNA was also capable of suppressing tumor cell migration by targeting Notch-1 signaling.

**Figure 4:**
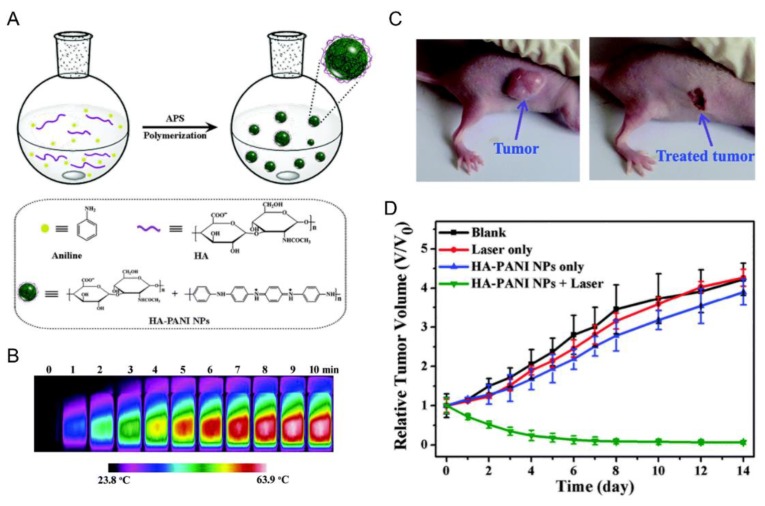
(**A**) Schematic illustration of the synthesis of HA-poly(aniline) (HA-PANI) nanoparticles. (**B**) Photothermal images of solution 100 mg /mL of HA-PANI in water upon exposure to 808 nm laser (2 W/cm^2^) from 0 to 10 min. (**C**) Representative photographs of the tumor and the treated tumor six days after injection of HA-PANI solutions and irradiation at 808 nm and 0.64 W/cm^2^. (**D**) Tumor growth rates of groups after different treatment. A saline solution of HA–PANI was injected into the tumor site and exposed to 808 nm laser (*n* = 3). Mice with no injection of HA–PANI s (laser only, n = 3) or without 808 nm laser exposure (HA–PANI only, n = 3); blank group with neither injection of HA–PANI nor 808 nm laser exposure (n = 3). The results are expressed as the mean ± SD (n = 3). Adapted with permission from [10]. Copyright 2019, Royal Society of Chemistry.

There are fewer examples found in literature of HA-based polyelectrolyte nanoparticles. In this sense, and with the aim of obtaining photothermal nanoparticles specifically targeted towards CD44 bearing cancer cells, Jing et al. [10] synthesized HA-poly(aniline) (PANI) nanoparticles. These nanocarriers are obtained by direct fabrication method, where aniline is polymerized, via oxidative polymerization in water in the presence of HA (Figure 4A). HA acts as anionic stabilizer that twist the synthesized PANI, due to electrostatic interactions, rolling both of them into random nanoparticles of ~100 nm. The interest of using PANI is its strong NIR absorption (generating substantial amount of heat energy) when its half-oxidized form is doped with an acid (emeraldine salt form), thus being a photothermal agent. Therefore, these nanoparticles are capable of heating upon exposure to 808 nm laser (Figure 4B). Furthermore, in vitro studies showed that they can selectively kill the cancer cells like HeLa (cervical cancer cells) and HCT-116 (colon cancer cells) rather than normal cells like human foreskin fibroblast (HFF) cells upon exposure to the laser. In vivo results confirmed the efficacy of the photothermal ablation of the tumor (Figure 4C,D).

### 4.3. pH-Responsive Nanocarriers

It is well known that the physiological pH in cancer cells is lower than that in blood and normal tissues. Furthermore, endosomal pH ranges between 6.5 and 4.5 [65]. Hence, the acid-triggered rapid release of drugs can be achieved inside tumor cells by using nanogels bearing pH-responsive polymers such as poly(l-histidine) (PHis) [57,58,59] or poly(diisopropylaminoethyl) aspartamide (PDIPASP) [60]. In addition to its biocompatibility and biodegradability, PHis shows a natural amphoteric property (deprotonation-protonation (pKa = 6.5) of its imidazole ring), while PDIPASP has an amino group that can be protonated-deprotonated when changing pH from 5 to 7.4. Another strategy relies on the use of synthetic polymers that can be hydrolyzed at pH 5.5. Thus, once in the lysosomes, the nanocarrier is degraded. Complexation of poly(β-amino ester) (PBAE) and HA produced nanocarriers that can easily encapsulate drugs at physiological pH but degraded at pH 5.5 thus favoring the release of the drug [24].

**Figure 5:**
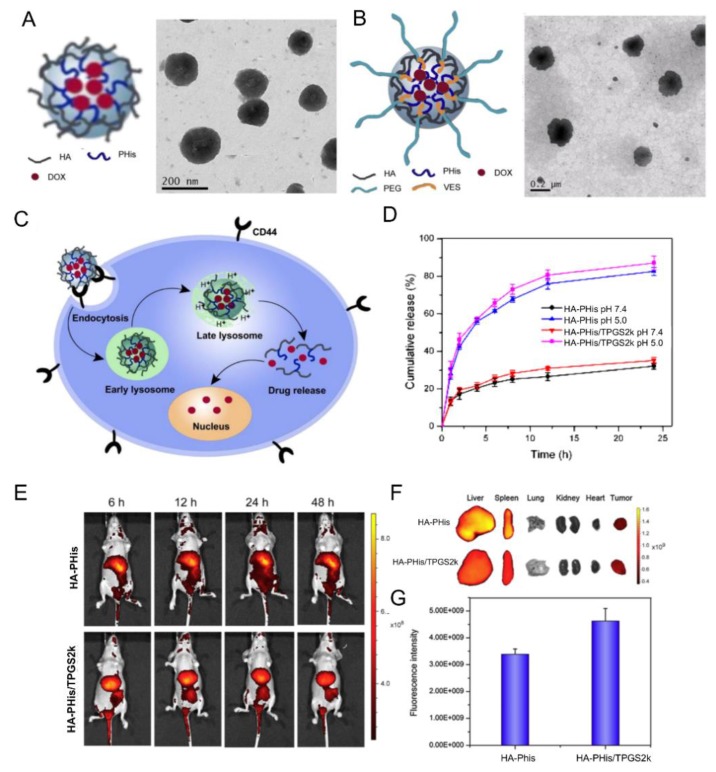
Schematic illustrations of self-assembly and TEM image of HA-PHis nanogels (**A**) HA-PHis/TPGS-2k (**B**). (**C**) pH-sensitive release profile of DOX from HA-PHis in PBS (pH 7.4 and 5.0) at 37 °C. Data as mean values ± SD (*n* = 3). (**D**) pH-sensitive release profile of DOX from HA-PHis and HA-PHis/TPGS2k in phosphate buffered saline (PBS) (pH 7.4 and 5.0) at 37 °C. Data as mean values ± SD (*n* = 3). (**E**) In vivo imaging of tumor-bearing mice administrated with DIR-loaded micelles. Images taken after administration of HA-PHis at 4 h and 12 h, and HA-PHis/TPGS2k for 4 h and 12 h, respectively. (**F**) Ex vivo fluorescence images of tumors and organs collected at 12 h post-injection of HA-PHis/TPGS2k and HA-PHis micelles. (**G**) Quantification of the ex vivo tumor uptake characteristics of micelles. Uptake expressed as fluorescence per mm^2^ of tumor. Data as mean values ± SD (*n* = 3). Adapted with permission from [57,58]. Copyright 2019, Elsevier.

Qui et al. demonstrated the possibility to prepare self-assembled nanogels based on HA-PHis and to induce pH-responsive intracellular drug delivery [57] (Figure 5A,B). They further exploited the potential of this system to overcome multidrug resistance (MDR). To do so, they synthesized a nanogel by mixing HA-PHis with a poly(ethylene glycol) (Mw 2000) modified with a d-α-tocopheryl molecule at the end (HA-PHis/TPGS2k). The aim was to encapsulate DOX in order to have pH-responsive nanogels that co-deliver DOX and TPGS2k into drug-resistant breast cancer MCF-7 cells (MCF-7/ADR) [58] (Figure 5). Tocopheryl demonstrated to inhibit the efflux pumps of P-glycoprotein 1 (P-gp, or multidrug resistant protein 1) leading to the restored sensitivity to anticancer drug [66]. Compared to HA-PHis, HA-PHis/TPGS2k nanogels showed higher cytotoxicity against the MCF-7/ADR cells. However, cytotoxicity of both nanocarriers was comparable for normal MCF-7 cells. The enhanced MDR reversal effect was attributed to the higher amount of cellular uptake of HA-PHis/TPGS2k in MCF-7/ADR cells than HA-PHis. The measurements of P-gp expression level indicated that HA-PHis/TPGS2k inhibited P-gp activity but without inhibition of P-gp expression. Moreover, in vivo studies indicated that HA-PHis/TPGS2k could reach the tumor site more effectively than HA-PHis. Thus, the pH-sensitive mixed nanogels system was shown to be a promising approach for overcoming the MDR. In order to improve targeting efficacy, HA-Phis/TPGS2k was decorated with a tumor cell-specific peptide ligand: the human epidermal growth factor receptor 2 (Her2) peptide [59]. In vivo assays demonstrated that the addition of the peptide to the nanocarriers produce a more efficient tumor killing activity.

HA-based nanogels bearing carboxylic containing polymers are also attractive carriers for anticancer drug delivery. In this case, the aim is not to trigger the release of the drug in the tumor cell, like nanocarriers from polymers containing basic groups, but to favor self-assembly at physiological pH (while being soluble at pH 10) as well as to achieve a sustained release. To this end, Kesharwan et al. [53] designed nanogels based on HA-poly(styrene-*co*-maleic anhydride) (HA-SMA). The author took advantage of the anhydride groups present in the polymer to produce HA-polymer conjugates by reaction with the hydroxyl groups of HA (Figure 2, Table 2). The esterification reaction is carried out at basic pH, which favors the hydrolysis of the unreacted anhydride group into the pH-responsive carboxylic acid groups. The authors chose 3,4-difluorobenzylidene curcumin (CDF) as an anticancer drug to test encapsulation and release. They observed that the release of CDF was much lower and sustained at acidic pH (5.5) compared to the release at basic pH (10), probably due to an increase of hydrophobicity. Nonetheless, from in vitro studies, the authors suggested that release of the drugs from these nanogels is driven by different factors including degradation, dissolution and/or diffusion. Furthermore, compared to SMA nanogels, HA-SMA nanogels exhibited a marked increase in the anticancer activity towards in CD44+ tumor cells.

### 4.4. Thermoresponsive Nanocarriers

Thermoresponsive copolymers have been advantageously used to prepare HA-copolymer conjugates that can self-assemble into nanogels at a given temperature (critical aggregation temperature, CAT). By varying the copolymer composition and molar mass, the CAT of the HA-copolymer derivative can be precisely tuned to obtain nanogels at the body temperature. Based on this, the group of Auzely-Velty et al. has synthesized a large variety of thermoresponsive HA based-nanogels [20,42,43,48,49].

First, they demonstrated that the grafting of a thermoresponsive copolymer, poly(diethylene glycol methacrylate-*co*-oligoethylene glycol methacrylate) (poly(DEGMA-*co*-OEGMA), onto HA allowed temperature-triggered assembly of HA-poly(DEGMA-*co*-OEGMA) into nanogels with a CAT of 34 °C. This copolymer was coupled with HA both via thiol-maleimide coupling [42] and radical thiol-ene addition reactions [48] (Table 2). These nanogels exhibited interesting features for drug delivery such as facile formation by simply heating the HA-copolymer solution, tunable size, easy loading of hydrophobic molecules, selectivity for cells expressing the CD44 receptor. In vivo biodistribution studies showed that the nanogels circulated freely in the blood but they were rapidly phagocytized within 13 min by circulating macrophage, limiting their therapeutic efficacy.

**Figure 6:**
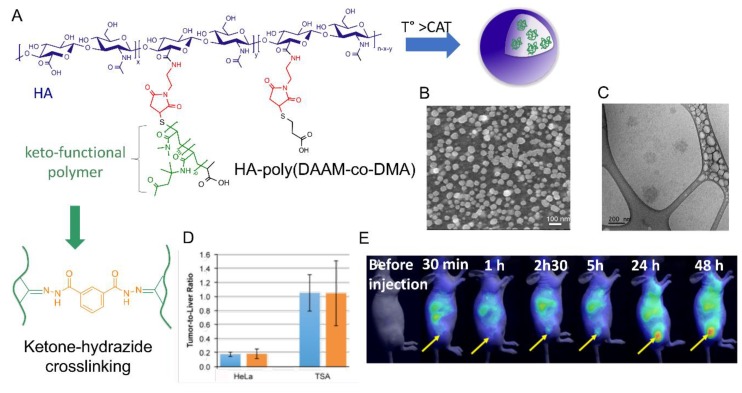
(**A**) Formation of hyaluronic acid-based nanogels by temperature-induced self-assembly and their covalent crosslinking by hydrazone bond formation within the hydrophobic domains of the grafted copolymer chains Morphology of HA-based nanogels crosslinked with an IDH:ketone molar ratio of 0.5 observed at 5 °C by TEM (**B**) and by cryo-TEM (**C**). (**D**) In vivo near-infrared Fuorescence (NIRF) images of the time dependent biodistribution of Cy5.5-labeled crosslinked nanogels in breast TS/A-pc and HeLa tumor-bearing mice. The tumor was engrafted subcutaneously on the right flank of the mouse. The fluorescence was measured before injection and at the following time elapse after administration: 30 min, 1h, 2 h 30, 5 h, 24 h, and 48 h. The tumor locations are indicated by the arrows. (**E**) Fluorescence intensity ratio of the excised tumor to liver at 24 h (blue) and 48 h (orange) post-injection. The results are expressed as the mean ± SD (*n* = 3). Adapted with permission from [20]. Copyright 2019, Royal Society of Chemistry.

The authors subsequently focused on the design of more sophisticated nanogels with increased colloidal stability after administration in vivo. They developed core-crosslinked nanogels based on HA modified with a copolymer of diacetone acrylamide (DAAM) and *N*,*N*-dimethylacrylamide (DAM) (poly(DAAM-*co*-DMA) [20]. The selective core-crosslinking of nanogels relied on the selective formation of hydrazone crosslinks with dihydrazides crosslinkers within the globular domains of the copolymer chains formed above the cloud point temperature (Figure 6). The efficiency of the keto-hydrazide ligation allowed tuning the crosslinking density by varying the dihydrazide crosslinker-to-ketone molar ratio. After core-crosslinking, the nanogel structure became “frozen” and remained stable at low temperature (below the CAT) with a size of 200 nm. To get new insight into the mechanism that determines accumulation of the nanogels in tumor tissues, in vivo biodistribution studies were carried out in two different mouse tumor models showing different degrees of EPR-mediated drug targeting as well as CD44 expression levels. These studies showed long circulation of nanogels in blood flow and gave evidence for their EPR effect-based tumor-specific targeting. Importantly, these experiments also demonstrated similar in vivo behavior of native HA in both tumor models, indicating that the nanogels biodistribution is mainly determined by the nature of the shell-forming polysaccharide.

Shell-crosslinked nanogels were also developed from HA-poly(DEGMA-co-BMA) possessing a CAT around 20 °C [49]. These soft nanocarriers were prepared in a very straightforward way using thiol-ene chemistry allowing (i) the coupling of the thiol-end functionalized copolymer with the polysaccharide modified with pentenoate groups and, (ii) the subsequent crosslinking of the shell-forming polysaccharide by reaction of a bi-functional thiol reagent with the remaining alkene groups (Figure 2). The authors analyzed the biodistribution in Ehrlich solid tumor-bearing mice for both the crosslinked and non-crosslinked nanogels. Interestingly, despite slight differences in the nanocarriers size (Table 2), no significant difference was found in the biodistribution of the nanogels.

### 4.5. Redox-Responsive Nanocarriers

Cancer cells have a reducing environment (defined as the amount of glutathione (GSH) present (20 mM)) compared to normal cell ([GSH] = 2–10 mM) or extracellular fluids ([GSH] = 2 – 20 µM) [67]. This unique intracellular redox potential enables the versatile design of redox-sensitive nanocarriers that release anticancer drugs in response to this change of redox environment. One of the best-known strategies consists of adding disulfide bonds in the nanocarriers as they can be reduced by GSH. Two different approaches have been developed for the incorporation the disulfide bonds in HA-based nanocarriers: (a) in the polymers or HA [34,36,37] and, (b) as a linker between the HA and the polymer [33,44,56]. The first strategy has been used for cross-linking the nanocarriers so that, once in the tumor cell, the bond is reduced and the nanocarrier disassembles. The second strategy confers on the system the ability, once in the tumor cells, to cleave the HA-polymer conjugate, resulting in the drug release.

**Figure 7:**
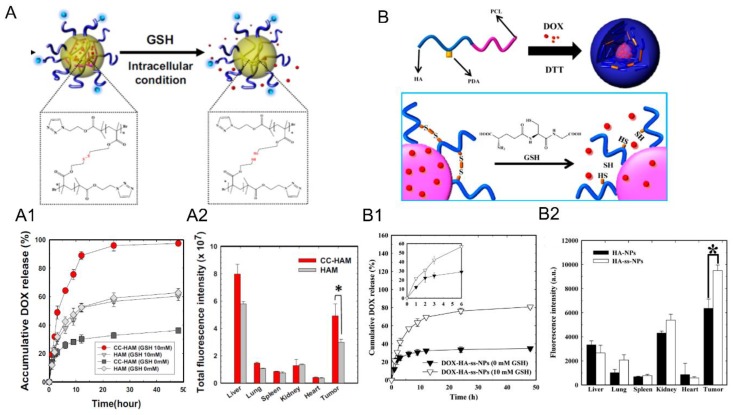
(**A**) Synthetic scheme of core crosslinked HA-b-poly(PDSMA) (CC-HAM) micelles loaded with DOX. (**A1**) Release profiles of DOX from CC-HAMs and HA-b-poly(PDSMA) (HAM) in the absence or the presence of GSH. The error bars in the graph represent standard deviations (*n* = 5). (**A2**) Fluorescence intensity of tumors and organs. Asterisks (*) denote statistically significant differences (* *p* < 0.05) calculated by one-way ANOVA test. (**B**) Synthetic scheme of the formation of DOX-loaded crosslinked micelles and their glutathione (GSH) responsive drug release behavior (**B1**) In vitro release behavior of DOX from DOX-HA-ss-NPs in the absence or the presence of GSH. The error bars in the graph represent standard deviations (*n* = 3). (**B2**) Quantification of the ex vivo tumor-targeting characteristics of HA micelles in tumor bearing mice. Error bars in the graph represent the standard deviation for five animals per group. Adapted with permission from [34,37]. Copyright 2019, American Chemical Society and Elsevier, respectively.

Han et al. selected the first approach to design two different micelles: one shell- [34] and the other core- [37] crosslinked. In both cases, they chose the same crosslinker precursor: pyridyldisulfide, (PDS). Disulfide crosslinks are formed after the addition of DTT, which can be cleaved by GSH in cancer cells. The core-crosslinked micelles (CC-HAM), based on HA-b-PDSMA (poly(pyridyldisulfide methacrylate) (Figure 7A), and shell-crosslinked micelles (HA-ss-NPs), based on HA-*b*-PCL (Figure 7B), were synthesized via click chemistry. A drug model, DOX, was encapsulated and micelles with a size around 200 nm were obtained for both systems. Interestingly, both micelles exhibited different release profiles of drug and biodistributions in SCC7 tumor-bearing mice. The release of DOX for core-crosslinked micelles was more efficient in the presence of GSH (100% versus 80%), while it was the same for both systems in the absence of GSH (~30%). Regarding the biodistribution profiles, the tumor-to-liver ratio indicated a higher accumulation of HA-ss-NPs in the tumor (tumor-to-liver ratio > 1) than CC-HAM (tumor-to-liver ratio < 1). Comparison between the crosslinked and non-crosslinked micelles (for each nanosystems) showed that the former exhibited higher structural stability in the presence of serum, sodium dodecyl sulfate or in the bloodstream. Furthermore, the crosslinked micelles showed a significant increase (up to a 30% more) of the tumor-to-liver ratio when compared to the non-crosslinked ones.

Polymersomes have also been shell-crosslinked by conjugating poly(trimethylene carbonate-co-dithiolanetrimethylenecarbonate), (P(TMC-*co*-DTC)) to a modified carbonyl group of HA [36]. Such an approach becomes appealing as it allows, in a one pot reaction, to graft a thiol-containing molecule like mertansine (DM1) toxin and, at the same time, to crosslink the polymersome due to the dithiolane group. In the presence of GSH, the drug is released and the polymersome becomes uncrosslinked. In vitro and in vivo analysis shows that the polymersomes are capable of inhibiting the growth of MDA-MB-231 human breast tumor with little adverse effect.

As discussed above, it is also possible to incorporate redox-cleavable bonds to trigger drug release. Hu et al. [44] used this approach to synthesize HA-SS-PLGA nanoparticles, composed of a hydrophobic PLGA chain and a hydrophilic HA chain linked through a cysteamine. With a double emulsion method, a nanosystem was constructed to deliver DOX and cyclopamine (CYC, an inhibitor of the hedgehog signaling pathway of cancer cells). CD44-overexpressing breast cancer stem cells and a bulk breast cancer cell were chosen to evaluate, in vivo and in vitro, the redox responsive release. A similar strategy was used by Park et al. [33] to create redox-responsive micelles (HA-ss-PLGA). In this case, cysteamine was used as a linker between the polymer (PGLA) and the carbonyl group. As expected, the release of encapsulated DOX is proportional to the concentration of GSH. Furthermore, the HA-ss-PLGA can target CD44-expressing tumor cells.

### 4.6. Light-Responsive Nanocarriers

Light-responsive nanocarriers differentiate from the previously described stimuli-responsive systems in that they alter their chemical and/or physical properties not upon exposure to internal stimuli (pH, redox state, enzyme triggers), but upon the application of an external one, such as light. [60] These nanocarriers have emerged as an attractive manner to induce drug release with spatial and temporal control by remote activation [68,69,70].

In this regard, Stefanello et al. [48] developed light- and thermoresponsive HA-based nanogels to trigger drug release “on-demand”. The strategy for their synthesis relied on the incorporation of light-cleavable units (coumarin ester derivatives) within a thermoresponsive ethylene glycol-based copolymer. The coumarin derivatives act as photoremovable protecting groups inducing a shift in the cloud point temperature of the grafted copolymer due to the conversion of coumarin esters into carboxylate groups upon light irradiation (near infrared or UV exposure). By grafting a copolymer based on diethylene glycol methacrylate (DEGMA) and coumarin methacrylate (CMA) onto HA (Table 2), the authors successfully obtained light and thermoresponsive nanogels whose CAT shifted from 27 °C (below human body temperature) to 38 °C (above human body temperature) after UV exposure. Furthermore, in vitro and in vivo biological studies revealed efficient internalization of the nanogels by cancer cells overexpressing the CD44 receptor of HA and their ability to circulate for a prolonged period of time in the bloodstream after intravenous injection in mice and considerable detection in tumor tissues.

**Figure 8:**
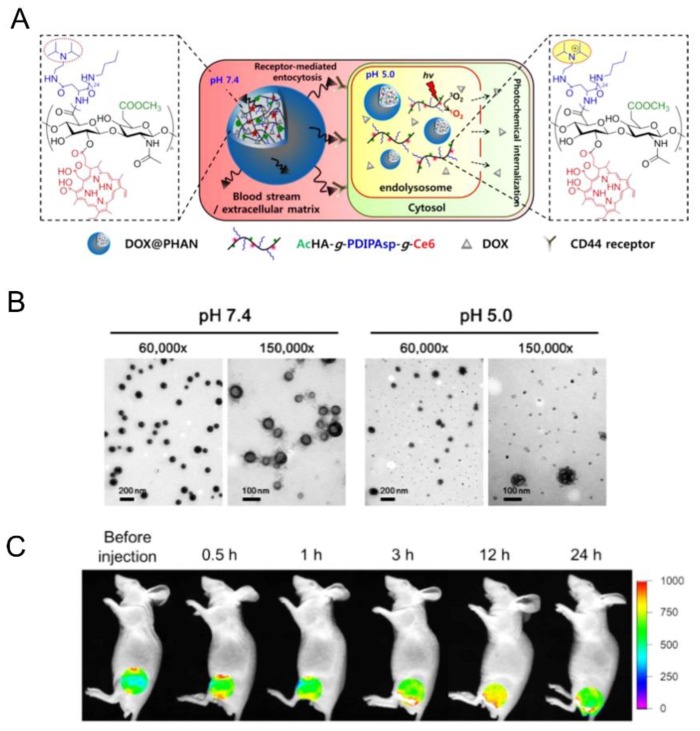
(**A**) Schematic representation of doxorubicin-loaded pH-responsive hyaluronic acid nanonogels (DOX@PHANs). (**B**) TEM photographs of DOX@PHANs at pH 7.4 and 5.0. Magnification is 6000× or 15,000×. (**C**) In vivo near-infrared fluorescence (NIRF) images of time-dependent accumulation of DOX@PHANs in CT-26 tumor-bearing mice after systemic administration via tail vein route. Adapted with permission from [60]. Copyright 2019, American Chemical Society.

On the other hand, Lee et al. [60] proposed to combine pH- and light-responsive properties (Figure 8). To this end, a photochemical agent, chlorin (Ce6) and a pH-responsive polymer, poly(diisopropylaminoethyl)aspartamide (PDIPASP) were grafted via a carbodiimide-mediated reaction to the carboxylate group of an acetylated HA (AcHA-*g*-PDIPASP-*g*-Ce6). The resulting nanogels, DOX@PHAN (size~200 nm and DOX loading content: 14%), displayed a pH-response activity, as mentioned in Section 4.3. Moreover, after cellular uptake via receptor-mediated endocytosis, intensity laser irradiation stimulated Ce6 grafted on HA to produce reactive singlet oxygen, which released DOX into the cytosol (Figure 8A).

## 5. HA-Based Polymeric Nanocarriers: Challenges and Future Prospects 

As it can be seen throughout this review, HA-based polymeric nanocarriers are an attractive system for biomedical applications, mainly anticancer therapy due to recognition of HA by CD44, which allows active targeting towards certain tumor cells. However, several in vivo studies showed that HA-based polymeric nanocarriers can also accumulate in the liver [20,46,49,52]. There are two possible explanations for this accumulation: the interaction of HA with the hyaluronan receptor for endocytosis (HARE) expressed by liver sinusoidal endothelial cells and the cellular uptake by phagocytic cells of the reticuloendothelial system [71]. In order to reduce this accumulation, surface modification with PEG of HA-based nanocarriers has been suggested to increase the tumor-to-liver ratio in comparison to bare HA nanocarriers [72,73,74]. However, the PEG/HA ratio in the hydrophilic shell is an important parameter to control in order to maintain HA binding to CD44. Furthermore, it should be pointed out that although adding PEG to a therapeutic cargo is a process approved by Food and Drug Administration (FDA), the synthetic nature of this polymer presents some drawbacks as some reports suggests that PEG can prompt antibody formation against PEG as well as trigger complement activation [75,76,77,78]. In this regard, there is still work to do to improve the biodistribution of HA-based polymeric nanocarrier without losing its ability to target tumors.

In addition to application in antitumor therapy, nanocarriers based on HA-polymer conjugates or complexes can also be used for several other applications, like rheumatoid arthritis (RA) therapy, delivery of drugs through the brain-blood barrier (BBB), siRNA delivery, sensing bacterial enzymes, imaging, among others [79]. However, as described in this review, only few examples of such applications have been investigated using HA-based polymeric nanocarriers, leaving a large field for future exploration. Imaging via encapsulation of fluorescent molecules and siRNA delivery have only been evaluated for these type of carrier when related to anticancer therapy. With regard to RA therapies, the possible application of HA-based polymeric nanocarriers is closely linked to the ability of HA to bind CD44, which is overexpress in synovial fibroblasts and tissue [80]. Another possible application of HA-based polymeric nanoparticles that has not been explored yet is the delivery of drugs through the BBB, mainly for brain cancer treatment, given HA ability to target these cells. It is a well-known fact that delivery of drugs to the brain is a major challenge due to the inability of drugs, mainly the hydrophilic ones, to cross BBB. Nanocarriers have come as a possible solution to this problem. In particular, polymeric nanosystems have proven to be capable of crossing this barrier, [5,7,32] thus making HA-based polymeric nanocarriers excellent candidates for therapies against glioblastoma.

## 6. Conclusions

In the past decade, several reviews have described the advantages and properties of polymeric nanocarriers [5,7,9,32,62,65,81] as well as HA-based nanosystems [6,79,82,83,84,85,86]. Nonetheless, to the best of our knowledge, none of them have focused on polymeric nanocarriers combining HA and custom-made polymers as major components, as done in this review. Based on the above-mentioned discussion, these type of nanosystems integrate the advantages provided by both types of macromolecules. HA provides hydrophilicity to the system, contributing to the stability of nanocarriers, especially those made of hydrophobic polymers. In addition, it confers biodegradability and biocompatibility. Furthermore, interaction with membrane-anchored proteins, like CD44, favors accumulation of HA-based nanocarriers in cancer cells, which makes anti-cancer therapy one of the most common applications of HA-based drug delivery systems. In addition, the presence of various functional groups in the HA backbone and its polyelectrolyte character offer a number of possibilities for engineering nanocarriers either by polyelectrolyte complexation or using conjugation methods allowing the synthesis of amphiphilic block or graft copolymers.

The synthetic polymers, on the other hand, can provide different properties depending on their structure. The selection of hydrophobic or hydrophilic polymers as well as neutral or positively charged ones, depends on the type of nanocarriers desired as well as the type of drug to be encapsulated (hydrophilic, hydrophobic or charged). Furthermore, biodegradable polymers are usually chosen as they allow the fabrication of fully degradable systems. Recent research has focused on using stimuli-responsive polymers for nanocarrier design as they allow to control assembly/ disassembly of the nanosystems. In this sense, it is possible to achieve selective release of drugs at the targeted site in response to a stimulus, which can be an internal change between the targeted cell and the rest of the body (mainly pH and redox potential) or an external source (light).

Thus, nanocarriers based on HA and tailor-made polymers are a promising field of research for finding nanotechnological solutions in nanomedicine, especially in anti-cancer therapy. Research in this area has only started as there is still many possible combinations of HA and copolymers as well as type of nanocarriers that have not been investigated yet. The promising results obtained thus far fully justify further development of new generations of HA-based polymeric nanosystems, especially stimuli-responsive biodegradable nanocarriers.

## Figures and Tables

**Table 1:** HA-*b*-polymers nanocarriers.

Mw of HA kg/mol	Polymer Structure ^a^	Size (nm) ^b^	DL (EE) %	In Vitro and/or in Vivo Biological Studies ^a^	Ref
5	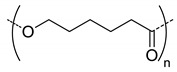 *PCL, biodegradable*	- ^c^	-	Detection of pathogenic bacteria “Staphylococcus aureus” and drug release of after enzymatic degradation.	[31]
2100		~200 ^d^			
12		198 ^e,f^	7 (74) ^f^	In vitro: cytotoxicity and intracellular drug release of DOX in SCC7 cell line. In vivo: biodistribution and antitumor efficacy in SCC7 cells-bearing mice.	[34]
		162 ^g,h^	9 (90) ^g^		
7.5	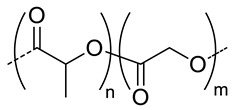 *PLGA, biodegradable*	<200 ^h^	8	In vitro: cytotoxicity in MDA-MB231 and NIH3T3 cell lines and release behavior of DOX. In vivo: biodistribution and antitumor efficacy in mice bearing MDA-MB231 or NIH3T3 cells grafted.	[33]
5	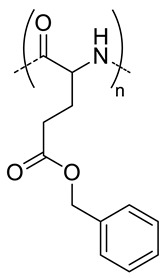 *PBLG, biodegradable*	220 ^d^	12 (40)	In vitro: cytotoxicity, cell uptake of DOX containing nanocarriers in MCF-7 and U87 cells lines. In vivo: antitumor efficacy in mice with DMBA induced tumor.	[29]
		135 ^d^	9.8 (49)	In vitro: cytotoxicity in MCF-7 and U87 cells lines and release behavior of DOC. In vivo: biodistribution in EAT–bearing mice.	[28]
		- ^i^	- ^i^	In vivo: stability, biodistribution, pharmacokinetics and antitumor activity of DOX containing nanocarriers in EAT-bearing mice.	[30]
		30 and 300 ^d^	-	In vitro and in vivo lung tumor cells targeting effect of the size.	[35]
8	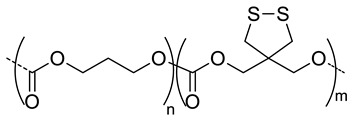 *P(TMC-co-DTC), biodegradable and redox-responsive*	103 ^d,g^	17 (99)	In vitro: cytotoxicity in MDA-MB-231 cell line and release behavior of DM1. In vivo: pharmacokinetics and anticancer efficacy in MDA-MB-231 cells-bearing mice.	[36]
7.4	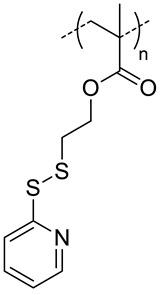 *PDSMA, redox-responsive*	215 ^f,h^ 187 ^g,h^	8 (80) ^f^ 9 (87) ^g^	In vitro: drug release of DOX. In vivo: biodistribution, pharmacokinetics, tumor accumulation profiles and antitumor efficacy in SCC7 cells-bearing mice.	[37]

^a^ abbreviations: PLC: poly(caprolactone), PLGA: poly(lactide-co-glycolide), PBLG: poly(benzyl-l-glutamate), P(TMC-co-DTC): poly(trimethylene carbonate-co-dithiolane trimethylene carbonate), PDSMA: poly(pyridyldisulfide methacrylate, DOX: doxorubicine, DOC: docetaxel, DM1: mertansine toxin DM1, DMBA: 7,12-dimethylbenz[a]anthracene; ^b^ without encapsulated drug; ^c^ only evaluated for nanocarriers with the drug encapsulated; ^d^ determined by dinamic light scattering (DLS); ^e^ determined by particle analyzer; ^f^ non crosslinked nanocarrier; ^g^ crosslinked nanocarrier; ^h^ determined by transmission electron microscopy (TEM); ^I^ authors inform that nanocarriers were obtained as described by Upadhyay et al. [29].

**Table 2:** HA-*g*-polymers nanocarriers.

Mw of HA kg/mol	Polymer Structure ^a^	Size (nm) ^b^	DL (EE) % ^a^	In Vitro and/or in Vivo Biological Studies ^a^	Ref
5.7	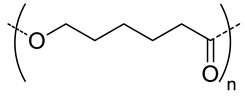 *PCL, biodegradable*	95 ^c^	(96)	In vitro: hemolytic toxicity and stability of DOX containing nanocarriers. In vivo: biodistribution and tumor inhibition in EAT-bearing mice.	[39]
**1500**	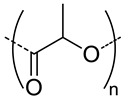 *PLA, biodegradable*	30 ^c^	5 (10)	In vitro: cytotoxicity and uptake in HCT-166-cells and release behavior of DOX.	[50]
8.3	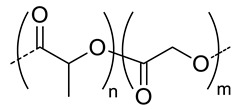 *PLGA, biodegradable*	103 ^c^	8	In vitro: cell viability and uptake in Hep G2 cells and CT26 cell lines and release behavior of DOX.	[51]
**15**		- ^d^	16 (87)	In vitro: cytotoxicity and uptake in Raw 264.7-cells and release behavior of SN38 In vivo biodistribution, and tumor inhibition in tramp-C1 cells–bearing mice.	[45]
5.7		152 ^e^	(80)	In vitro: cytotoxicity in EAT-cells and release behavior of 5-FU In vivo: biodistribution, and tumor inhibition in EAT–bearing mice.	[46]
6.4		245 ^c^	(71) DOX (58) CYC	In vitro: cell viability and uptake in MCF-7 and MDA-MB-231 cell lines and release behavior of DOX and CYC. In vivo: synergic antitumor efficacy of DOX and CYC in MDA-MB231 cell-bearing mice.	[44]
5.7	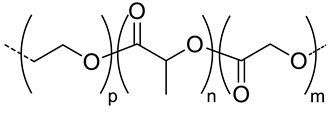 *PEG-co-PLGA, biodegradable*	- ^d^	(~90)	In vitro: drug release of DOX. In vivo: biodistribution, and tumor inhibition in EAT–bearing mice.	[52]
10	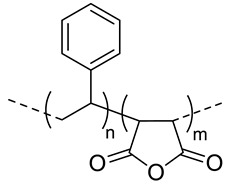 *SMA, pH-responsive after hydrolysis of anhydrides*	- ^d^	(16)	In vitro: cell viability and uptake of CDF containing nanocarriers in MiaPaCa-2 and AsPC-1; activity on CD44+ and CD44- pancreatic cells.	[53]
20	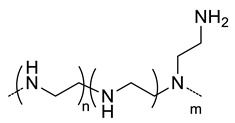 *Branched PEI, positive charged at pH 7.4*	~200 ^c,f^	-	In vitro: siRNA release, cell uptake and gene silencing in MDA-MB468 cell line. In vivo: tumor uptake and gene-silencing in A549 cells-bearing mice.	[54]
		~100 ^c,g^	-	In vivo: biodistribution and quantification of siRNA in mice bearing A549, A549^DDP^, H69 or H69Ar cells grafted.	[55]
		~200 ^c,f^	33 (86)	In vitro: cytotoxicity, cell uptake and gene silencing of siRNA and PTX-containing nanocarriers in MDA-MB-231 cell line. In vivo: biodistribution and antitumor efficacy in MDA-MB-231 cells-bearing mice.	[56]
**- ^g^**	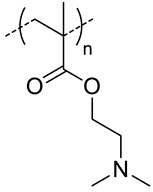 *pDEAEMA, positive charged at pH 7.4*	155 ^c^	-	In vitro: cytotoxicity in HCT 116 cells and uptake of siRNA.	[40]
11	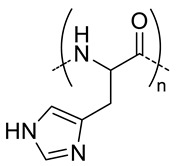 *pHis, pH-responsive*	~400 ^c^ (pH = 7.4)	7 (90)	In vitro: cell viability and uptake in MCF-7 cell line; endocytosis inhibition. pH release behavior of DOX containing nanocarriers.	[57]
			10 (92)	In vitro: cell viability and uptake in MCF-7 and MCF-7/ADR cell lines; endocytosis inhibition. pH release behavior of DOX and tocopheryl-PEG containing nanocarriers. In vivo: biodistribution in MCF-7/ADR cells–bearing mice.	[58]
		- ^d^	10 (93)	In vitro: cytotoxicity and cellular uptake in MDA-MB-231 cell lines. pH release behavior of DOX and Her2 peptide-tocopheryl-PEG containing nanocarriers. In vivo: biodistribution and antitumor efficacy in MDA-MB-231 cell-bearing mice.	[59]
5.8	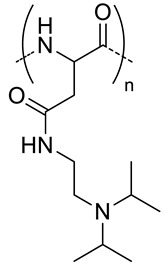 *PDIPASP, pH-responsive*	- ^d^	14	In vitro: cell viability and uptake in CT-26 cell line; photosensible endosome scape; pH release behavior of DOX. In vivo: biodistribution and antitumor efficacy in CT-26 cell-bearing mice.	[60]
**120**	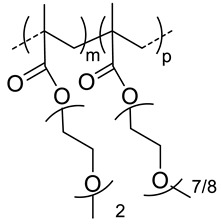 *p(DEGMA-co-OEGMA), thermosensible (CAT of HA-copolymer: 34 °C)*	150 ^c^ (40 °C)	~3 (70)	In vitro: cellular uptake by RAW264.7 macrophages of DSB containing nanocarriers. In vivo: biodistribution, and macrophage uptake in mice.	[48]
**300**		211 ^c^ (40 °C)	-	-	
200		95 ^c^ (40 °C)	(80)	In vitro: cell viability of PTX containing nanocarriers in HCT-8/E11 and SKOV-3 cell lines.	[42]
**40**	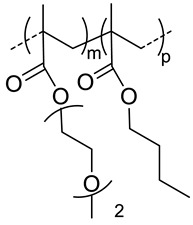 *p(DEGMA-co-BMA), thermosensible*	108 ^c,i^ (37 °C) 151 ^c,j^ (37 °C)	-	In vitro: vero cells viability. In vivo: biodistribution in EAC cells-bearing mice.	[49]
40	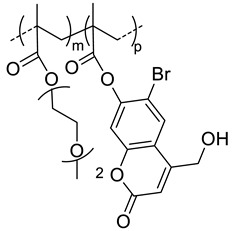 *p(DEGMA-co-CMA), photo and thermosensible*	~100 ^c^ (37 °C)	1.5 (52)	In vitro: cell viability in Vero cells and uptake in HeLa cells of PTX containing nanocarriers. In vivo: biodistribution in mice bearing HeLa subcutaneous grafts.	[43]
40	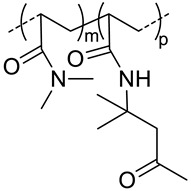 *p(DAAM-co-DMA), thermoresponsive*	144 ^c,i^ (40 °C) 150 ^c,j^ (40 °C)	-	In vitro: cell viability and uptake in HeLa and TS/A-pc cell lines. In vivo: biodistribution in mice bearing HeLa or TS/A-pc subcutaneous grafts.	[20]

^a^ abbreviations: PLA: Poly(lactide), PEG: polyethylene glycol, SMA: poly-(styrene-co-maleic anhydride, PEI: poly(polyethyleneimine), pDEAEMA: poly-N,N-diethylaminoethylmethacrylate, pHis: poly(histidine), PDIPASP: poly(diisopropylaminoethyl)aspartamide, p(DEGMA-co-OEGMA): poly(diethylene glycol methacrylate-co-oligoethylene glycol methacrylate), p(DEGMA-co-BMA): poly(diethylene glycol methacrylate-co-butylmethacrylate), p(DEGMA-co-CMA): poly(diethylene glycol methacrylate-co-coumarin methacrylate)), p(DEGMA-co-CMA): poly (diacetone acrylamide-co-N,N-dimethylacrylamide, CAT: critical aggregation temperature, SN38: 7-ethyl-10-hydroxylcamptothecin, 5-FU: 5-fluorouracil, CYC: cyclopamine, CDF: 3,4-difluorobenzylidene curcumin**,** siRNA: small interference RNA, PTX: paclitaxel, DBS: distrylbenzene derivative, Her2: human epidermal growth factor receptor 2.; ^b^ without encapsulated drug; ^c^ determined by DLS; ^d^ only evaluated for nanocarriers with the drug encapsulated; ^e^ determined by laser diffraction particle size analyzer; ^f^ nanoparticle formed when complexed with siRNA; ^g^ nanoparticle formed when complexed with siRNA and HA-PEG; ^h^ hydrolyzed from HA Mw 1058 kg/mol; ^i^ non crosslinked nanocarrier; ^j^ crosslinked nanocarrier.

**Table 3:** Nanocarriers based on HA-polymer complexes.

Mw of HA kg/mol	Polymer Structure	Size ^a^ in nm (T, °C)	DL (EE) %	In Vitro and/or in Vivo Biological Studies ^a^	Ref
- ^b^	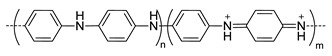 *Polyaniline, esmeraldine salt, positive charged, photothermal*	100 ^c^	- ^d^	In vitro: cytotoxicity and photothermal therapy effect in HFF, HCT-116 and HeLa cell lines. In vivo: photothermal therapy in HeLa tumor-bearing mice.	[10]
170	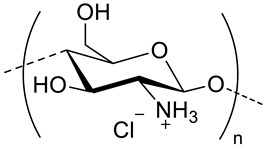 *Chitosan hydrochloride salt, biodegradable, positive charged*	189 ^c,e^	(48, DOX) (91, miR-34a)	In vitro: cytotoxicity and uptake of DOX and miR-34a containing nanocarriers in MDA-MB-231 cell line. In vivo tumor inhibition in MDA-MB-231 cells-bearing mice.	[22]
1300-1800	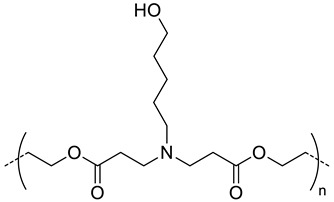 *Poly(β-amino ester), biodegradable, partially positive charged at pH 7.4*	- ^f^	- ^b^	In vitro: cell viability and uptake in MDA-MB-231 cells.	[24]
54.3 ^g^	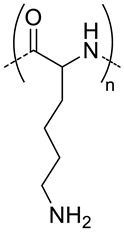 *Polyd -lysine, biodegradable positive charged at pH 7.4*	122 ^c,h^ 145 ^c,i^	- ^d^	-	[23]

^a^ without encapsulated drug; ^b^ data not informed; ^c^ determined by DLS; ^d^ no drug encapsulated; ^e^ for a weight ratio 2:1 HA:CS; ^f^ only informed for polyelectrolyte nanocarriers containing the drug; ^g^ HA was graft to a 5kDa NH_2_-PEG; ^h^ non crosslinked nanocarrier; ^i^ crosslinked nanocarrier.
